# Assessment of Hemoglobin A1c Management and Prescription Cost Due to Polypharmacy Among Patients With Diabetes in Iran Based on the STEPS Iran 2016 Survey and a Prescription Database: A Multi-level, Cross-sectional National Study

**DOI:** 10.34172/aim.2024.01

**Published:** 2024-01-01

**Authors:** Mojdeh Daneshmand, Hamidreza Jamshidi, Mohammad Hadi Farjoo, Mohammad Reza Malekpour, Erfan Ghasemi, Seyede Salehe Mortazavi, Mohsen Shati, Farshad Farzadfar

**Affiliations:** ^1^Department of Pharmacology, School of Medicine, Shahid Beheshti University of Medical Sciences, Tehran, Iran; ^2^Non-Communicable Diseases Research Centre, Endocrinology and Metabolism Population Sciences Institute, Tehran University of Medical Sciences, Tehran, Iran; ^3^Geriatric Mental Health Research Center, School of Behavioural Sciences and Mental Health, Iran University of Medical Sciences, Tehran, Iran; ^4^Mental Health Research Centre, Department of Epidemiology, Psychosocial Health Research Institute, Iran University of Medical Sciences, Tehran, Iran; ^5^Non-Communicable Diseases Research Centre, Tehran University of Medical Sciences, Tehran, Iran

**Keywords:** Cost, Diabetes, Hemoglobin A1c, Polypharmacy

## Abstract

**Background:**

Diabetes frequently results in the need for multiple medication therapies, known as ‘Polypharmacy’. This situation can incur significant costs and increase the likelihood of medication errors. This study evaluated the prescriptions of patients with diabetes regarding polypharmacy to assess its effect on the control of hemoglobin A1c (HbA1c) levels and prescription costs.

**Methods:**

A cross-sectional national study was conducted based on data from linking the Iranians Health Insurance Service prescriptions in 2015 and 2016 with the STEPS 2016 survey in Iran. The association of the individual and sociodemographic factors, as well as polypharmacy, as independent variables, with control of HbA1c levels and the cost of the prescriptions were assessed among diabetic patients using logistic and linear regression, respectively.

**Results:**

Among 205 patients using anti-diabetic medications, 47.8% experienced polypharmacy. The HbA1c of 74 patients (36.1%) was equal to or less than 7, indicating controlled diabetes. HbA1c control showed no significant association with gender. However, prescription costs were notably lower in females (β=0.559 [0.324‒0.964], *P*=0.036). No significant correlation was found between the area of residence and prescription costs, but HbA1c was significantly more controlled in urban areas (OR=2.667 [1.132‒6.282], *P*=0.025). Prescription costs were significantly lower in patients without polypharmacy (β=0.211, [0.106‒0.423], *P*<0.001), though there was no significant association between polypharmacy and HbA1c levels.

**Conclusion:**

Our results demonstrated that diabetics with polypharmacy paid significantly more for their prescriptions without experiencing a positive effect on the control of HbA1c levels.

## Introduction

 Metabolic syndrome (MetS), characterized by a combination of dyslipidemia, hypertension, and diabetes, poses a global health challenge. As of 2020, the prevalence of MetS was reported to be 4.8% among the global adult population.^1^ Its prevalence varies across populations and criteria, ranging from 8% in individuals with type 1 diabetes mellitus to as high as 80% in those with type 2 diabetes mellitus. Different factors and criteria contribute to these diverse prevalence rates.^2^ As of 2021, the global prevalence of diabetes in individuals aged 20‒79 was reported to be 536.6 million. Projections indicate that this figure is expected to increase, reaching 783.2 million patients by 2045. These estimates underscore the growing impact of diabetes on a global scale.^3^ In addition, 5 million deaths (18- and 99-year-old patients) related to diabetes were recorded worldwide in 2017, and 850 billion dollars were spent for diabetes worldwide in the same year, which is expected to rise to 958 billion dollars by 2045.^4^ In 2021, 14.15% of > 18-year-old patients in Iran had diabetes, and it is predicted to rise to 9.2 million patients by 2030.^5,6^ Physicians commonly prescribe multiple medications for diabetes treatment for two primary reasons, including the imperative for precise control of blood glucose, which often requires a combination of medications, and the presence of comorbidities such as dyslipidemia, cardiovascular diseases, renal failure, neuropathy, retinopathy, diabetic ulcers, obesity, and gastrointestinal diseases. The multifaceted nature of diabetes and its associated conditions necessitates a comprehensive approach to management, often involving a combination of medications to address various aspects of the disease and its complications.^7^ Concomitant use of 5 medications or more in a prescription, regardless of their clinical indication, is called polypharmacy.^8-11^ While polypharmacy itself is not inherently harmful, it does elevate the risk of potential issues such as drug-drug interactions, adverse drug reactions, patient non-compliance, and medication errors. These adverse consequences can contribute to a diminished quality of life, heightened morbidity and mortality, and place a financial burden on both patients and the healthcare system. The careful management of polypharmacy is crucial to mitigating these risks and optimizing the overall effectiveness of treatment regimens.^12^ Indeed, distinguishing adverse drug reactions from symptoms of the underlying disease can be challenging. This difficulty in differentiation raises the risk of a prescription cascade, a situation where patients end up taking additional medications to address the unintended reactions caused by their previous medications. This cycle can lead to a compounding effect, potentially exacerbating the complexity of the treatment regimen and further complicating the management of both the original condition and the drug-induced reactions.^13^ Hence, individuals dealing with polypharmacy encounter several challenges in medication adherence and the financial burden associated with both short-term medication expenses and the long-term consequences of polypharmacy, such as multiple healthcare facility visits and hospital admissions. These factors contribute significantly to the overall costs of the health system. Furthermore, given the considerable rise in medication costs due to inflation in Iran, it becomes crucial to evaluate the impact of polypharmacy on diabetes control, including assessing parameters such as hemoglobin A1c (HbA1c) levels in diabetic patients.^14-17^

## Materials and Methods

 This is a national, cross-sectional, multi-level study based on the STEPS method, which is the World Health Organization STEPwise approach to risk factor surveillance and is a set of feasible and standardized methods for data collection, processing, and reporting on a global scale.^18^

 The STEPS Iran 2016 survey is a national, large-scale, cross-sectional study that has assessed risk factors of 4 non-communicable diseases (cardiovascular, diabetes, cancers, and chronic respiratory diseases) in 389 districts of Iran and is a representative sample of the population at the national level. About 31 000 patients aged 25 years and older were enrolled in the STEPS 2016 study, and all their demographic and epidemiologic data, as well as risky behaviours, were recorded in the form of laboratory data, physical examinations, and questionnaire.^19^ In this study, based on patients’ unique national IDs, data from the STEPS Iran 2016 survey were linked with data from 102 million prescriptions related to Iranians Health Insurance Service (*Bime -Salamat*) records in 2015 and 2016, and about 16 000 patients were selected accordingly. Subsequently, individuals with MetS were selected based on the criteria established by the American Heart Association (AHA). According to the AHA, any individual meeting three or more of the following conditions—elevated triglyceride levels, hypertension, hyperglycemia, low levels of high-density lipoprotein, and a waist circumference exceeding > 102 cm in men or > 89 cm in women—is classified as a MetS patient.^20,21^ In this study, due to a lack of data regarding patients’ waist circumference, this item was not considered in defining criteria. From the pool of MetS patients, individuals with diabetes mellitus were chosen based on their fasting blood glucose levels. Next, only those patients who had prescriptions containing antidiabetic medications were included in our study. To ensure representation, one prescription was randomly selected and evaluated for each patient in each year of the study. This method aimed to capture a diverse and representative sample from the identified population. All medications in the prescriptions were recorded as anatomical therapeutic chemical codes in the database.^22^

 Polypharmacy was defined as prescriptions including 5 or more medications.^9-11^ To evaluate polypharmacy, only medications that appeared three times or more in prescriptions within one year were taken into consideration. This criterion was applied to specifically focus on medications that were consistently prescribed and used over the year, providing a more nuanced understanding of the polypharmacy phenomenon within the studied population.^23^ Individuals who experienced polypharmacy either in 2015, 2016, or in both years were classified into the “with polypharmacy” group for the purposes of this study. This inclusive approach ensured that individuals with a history of polypharmacy in either or both of these years were appropriately identified and categorized for analysis.

 All patients were categorized according to gender (male or female) and area of residence (urban or rural). Additionally, four age groups were defined, including 25‒39 years, 40‒59 years, 60‒80 years, and over 80 years old. The education level was stratified into four categories (illiterate, 1‒6 years of education, 7‒12 years of education, and more than 12 years). Furthermore, principle component analysis was already used in the STEPS project to define the wealth index of the patients.^24^ Patients were accordingly divided into 5 groups, including the poorest (0 ‒ < 20%), poor (20% ‒ < 40%), average (40% ‒ < 60%), rich (60% ‒ < 80%), and richest (80%‒100%) based on the percentage of their wealth index.^25^ According to the American Diabetes Association, 7% is the standard cut-off for HbA1c.^26^ Patients with an HbA1c level equal to or less than 7% were placed in the controlled group, and those with an HbA1c level higher than 7% were considered the uncontrolled group. The study involved calculating the cumulative cost of prescriptions for each patient in 2015 and 2016. Then, the association between polypharmacy and HbA1c levels was evaluated, as well as the relationship between polypharmacy and the associated costs. Specifically, the analysis aimed to understand the connection between the incurred cost and HbA1c levels. The cost was defined as the actual price of the prescribed medications listed in each patient’s prescription. This comprehensive approach allowed for an exploration of the financial implications of polypharmacy and its potential impact on diabetes control, as reflected in HbA1c levels.

 Analysis was performed based on both individual (gender and age) and sociodemographic (education level, area of living, and wealth index) factors. The study assessed the association between HbA1c levels and various factors, including polypharmacy, using unadjusted and adjusted logistic regression models. In one approach, HbA1c served as the dependent variable, while individual and socio-demographic factors, and polypharmacy were considered independent variables. Logistic regression was employed for this analysis. In another approach, the cumulative cost in 2015 and 2016 was taken as the dependent variable. Individual, socio-demographic factors, and polypharmacy were regarded as independent variables, and linear regression was applied for analysis. To address the non-normal distribution of the cost, the logarithm of the cost was calculated for assessments. The analysis was conducted using SPSS (version 22) and Python programming (version 3). A significance level of *P* ≤ 0.05 was considered statistically significant in the interpretation of the results.

## Results

 Among the 2075 MetS patients in 2015 and 2016, 510 had diabetes mellitus, and out of these, 205 patients (9.9%) were using blood sugar-lowering agents. Within this subgroup, 137 individuals (66.8%) were female, and 68 (33.2%) were male. Among these patients, 98 (47.8%) were identified as having polypharmacy. Notably, 74 patients (36.1%) exhibited an HbA1c level equal to or less than 7, indicating controlled diabetes.

 Regarding the cumulative cost of prescriptions in 2015 and 2016, it amounted to 58 800 442 and 69 381 530 rials for patients without and with polypharmacy, respectively. The analysis revealed several associations within the studied population:

###  Gender

 Control of HbA1c was not significantly associated with gender; however, the prescription cost was significantly lower in female patients (β = 0.559 [0.324‒0.964], *P* = 0.036).

###  Age

 No significant association was found between age and the control of HbA1c. Prescription costs were significantly higher in patients aged > 80 years (*P* = 0.003).

###  Area of Living

 There was no significant association between the area of living and the cost of prescriptions; however, HbA1c was significantly more controlled in urban areas (OR = 2.667 [1.132‒6.282], *P* = 0.025).

###  Education Level and Wealth Index

 Higher education levels showed insignificant associations with both higher prescription costs and better control of HbA1c levels.

 No significant association was observed the between wealth index and HbA1c levels, and there was no significant difference in cost between different wealth groups.

###  Polypharmacy

 Prescription costs were significantly higher in patients with polypharmacy (β = 4.73, *P* < 0.001).

 Controlled HbA1c was 36.7% in patients with polypharmacy and 35.5% in those without polypharmacy. However, no significant association was found between polypharmacy and HbA1c levels (Tables 1 and 2).

**Table 1:** Association Between Individual and Sociodemographic Factors, Polypharmacy, and HbA1c in Diabetes Mellitus Patients

**Factor**	**No. (%)**	**Controlled HbA1c** ** No. (%)**	**Crude OR**^a^ **(95% CI)**	***P*** ** Value**	**Adjusted OR**^a^ **(95% CI)**	***P*** ** Value**
Gender						
Female	137 (66.8)	44 (32.1)				
Male	68 (33.2)	30 (44.1)	1.669 (0.918–3.035)	0.093	1.405 (0.694–2.844)	0.344
Age group						
25–39	7 (3.4)	3 (42.9)				
40–59	80 (39.0)	26 (32.5)	0.642 (0.134–3.081)	0.580	0.554 (0.062–4.917)	0.596
60–80	108 (52.7)	41 (38.0)	0.816 (0.174–3.831)	0.797	0.666 (0.075–5.872)	0.714
> 80	10 (4.9)	4 (40.0)	0.889 (0.125–6.310)	0.906	1.237 (0.092–16.538)	0.873
Area of living						
Rural	44 (21.5)	9 (20.5)				
Urban	161 (78.5)	65 (40.4)	2.633 (1.186–5.844)	0.017*	2.667 (1.132–6.282)	0.025*
Years of schooling						
0	60 (29.3)	24 (40.0)				
1–6	64 (31.2)	23 (35.9)	0.841 (0.407–1.740)	0.641	0.887 (0.404–1.947)	0.765
7–12	52 (25.4)	12 (23.1)	0.450 (0.197–1.028)	0.058	0.378 (0.151–0.947)	0.038*
> 12	29 (14.1)	15 (51.7)	1.607 (0.658–3.925)	0.298	1.440 (0.548–3.785)	0.459
Wealth index						
Poorest	51 (24.9)	17 (33.3)				
Poor	42 (20.5)	16 (38.1)	1.231 (0.525–2.887)	0.633	1.446 (0.576–3.628)	0.433
Average	34 (16.6)	11 (32.4)	0.957 (0.379–2.412)	0.925	1.325 (0.477–3.683)	0.589
Rich	48 (23.4)	15 (31.3)	0.909 (0.391–2.113)	0.825	0.919 (0.360–2.346)	0.860
Richest	28 (13.7)	14 (50.0)	2.000 (0.780–5.131)	0.149	1.655 (0.605–4.524)	0.326
Polypharmacy						
No	107 (52.2)	38 (35.5)				
Yes	98 (47.8)	36 (36.7)	1.054 (0.596–1.865)	0.856	0.856 (0.448–1.637)	0.639

*Note*. ^*^ Indicates significance of the *P*-value; ^a^OR: Odds ratio; CI: Confidence interval; HbA1c: Hemoglobin A1C.

**Table 2:** Association of Prescription Cost With Individual and Sociodemographic Factors, and Polypharmacy

**Factor**	***P*** ** Value**	**Beta Coefficient (β)**	**CI (95%)**
Gender			
Female	0.036*	0.559	0.324–0.964
Male			
Age group			
25–39	0.228	0.647	0.318–1.314
40–59	0.001*	0.336	0.173–0.653
60–80	0.003*	0.502	0.319–0.791
> 80			
Area of living			
Rural	0.357	0.792	0.481–1.302
Urban			
Years of schooling			
0	0.251	0.694	0.372–1.295
1–6	0.011*	0.320	0.133–0.769
7–12	0.050*	0.439	0.192–1.001
> 12			
Wealth index			
Poorest	0.191	1.386	0.850–2.281
Poor	0.061	0.439	0.186–1.036
Average	0.795	0.933	0.550–1.581
Rich	0.666	0.888	0.519–1.520
Richest			
Polypharmacy			
No	< 0.001*	0.211	0.106–0.423
Yes			

*Note*. ^*^Indicates significance of the *P* value; CI: Confidence interval.

## Discussion

 In this comprehensive cross-sectional, multi-level, national study, our primary objective was to investigate the potential associations between polypharmacy, individual factors, sociodemographic factors, and the control of HbA1c levels in patients with diabetes mellitus during 2015 and 2016. Additionally, it was aimed at assessing the influence of these factors on the cost of prescriptions for these patients.

 The findings revealed a significant improvement in the control of HbA1c levels in urban areas compared to rural areas. However, there was no significant difference in medication costs between these two settings, indicating uniformity in prescription guidelines across urban and rural regions. This aligns with the findings of a study by Govan et al conducted in Scotland, encompassing 23 thousand patients with type 1 diabetes. Their research demonstrated a noteworthy association between HbA1c levels and the area of residence. Specifically, patients in more deprived areas exhibited higher HbA1c levels and experienced increased hospital admissions due to diabetic ketoacidosis.^27^ This consistency in results underscores the importance of considering geographic and socioeconomic factors in diabetes management and healthcare planning.

 The results of the current study indicated that patients with higher educational levels and greater wealth insignificantly tended to have better control of HbA1c levels, supporting the idea that socioeconomic factors play a role in health outcomes. The lack of a significant association between cost and education level or wealth status suggests that there might be a consistent protocol in place, ensuring equitable access to medications across different socioeconomic groups. The fact that wealthier individuals might not necessarily pay more for their prescriptions but still achieve better disease control underscores the multifaceted nature of healthcare disparities.

 The insight that better wealth status may be linked to improved living conditions, higher education levels, and enhanced access to healthcare facilities aligns with the broader understanding that addressing health disparities involves comprehensive improvements in infrastructure, education, and healthcare accessibility. The reference to a study in Madrid, where the prevalence of poor diabetes control was lower in medium and high neighborhood socioeconomic status, further emphasizes the impact of socioeconomic factors on health outcomes. It suggests that improving health in rural areas may require a holistic approach that includes educational system development and enhanced healthcare infrastructure rather than purely financial or monetary interventions.^28^ Our study’s findings on the lack of a significant association between age and gender and HbA1c levels conform to the results of a study in Sweden by Mellergård et al. It is interesting to note that, in both studies, age and education level did not show significant associations with HbA1c levels. The parallel findings in the Swedish study, where male patients exhibited more variability in their HbA1c levels, hint at potential gender-specific considerations in diabetes management. This underscores the importance of addressing not only clinical factors but also broader societal and economic factors when designing interventions for diabetes care.^29^ The noteworthy observation in the current study that male patients paid significantly more for their prescriptions, despite no significant difference in HbA1c control compared to female patients, suggests a potential disparity in the financial burden associated with diabetes management. It may be because men, due to having more job opportunities, a higher income, and better insurance coverage, bear a higher prescription cost. This emphasizes the complex interplay between socioeconomic factors and healthcare costs, especially in the context of chronic conditions such as diabetes.

 Our findings regarding the lack of a significant association between polypharmacy and HbA1c levels, but a significant increase in medication cost with polypharmacy, indicate a complex relationship between the number of medications and glycemic control. The observation that taking more medications might not necessarily lead to better control of HbA1c levels resonates with the notion that managing multiple medications brings its own set of challenges, including potential interactions and side effects. The reference to a systematic review and meta-analysis by Al-Musawee et al on over 1 million patients in Portugal in 2019 provides additional support for our findings. The absence of a significant association between polypharmacy and HbA1c control corroborates our study’s results. However, the significant positive association between polypharmacy and mortality highlights the importance of considering the broader impact of multiple medications on patient outcomes beyond glycemic control alone.^14^ The study by Patel et al, focusing on patients aged 65 and older in the United States, adds an interesting dimension to the discussion on polypharmacy and glycemic control. The division of patients into three groups based on the number of medications in their prescriptions—low, average, and high—provides a nuanced perspective. The notable finding that control of HbA1c was significantly better in the average group (5‒10 drugs) after two years of monitoring demonstrates a potential optimal range for medication complexity in the elderly population. This finding is in conformity with the idea that a moderate number of medications may be associated with better glycemic control compared to both lower and higher medication burdens.^30^ Understanding the ideal balance between medication management and glycemic control is crucial, especially in older populations where multiple chronic conditions are often present. It emphasizes the importance of personalized medicine and tailoring treatment plans to individual patient needs and complexities.

 It is important to note that the sample of population in the current study was a sub-sample of the STEPS survey; therefore, the results will be representative at the national level, although for more deductive results, it is suggested to perform further studies on larger populations.

## Conclusion

 The current study results provide insights into both the control of diabetes based on HbA1c levels and the associated medication costs, highlighting the impact of polypharmacy on the financial aspects of healthcare. These findings generally underscore the need for a holistic approach to medication management, especially in patients with complex conditions such as MetS. Balancing the benefits of multiple medications with the potential risks and challenges they pose is a critical consideration in optimizing overall patient care and outcomes. Considering the inflation and the rising trend of medication cost in Iran, it is essential to implement retraining programs for the physicians and pharmacists and to screen the prescriptions using online monitoring platforms and references, such as Beers criteria and Lexi-interact, to minimize the unnecessary polypharmacy, reduce unnecessary medication cost, and enhance patients’ quality of care in Iran.^7,31,32^
